# Intranasal Delivery of Galanin 2 and Neuropeptide Y1 Agonists Enhanced Spatial Memory Performance and Neuronal Precursor Cells Proliferation in the Dorsal Hippocampus in Rats

**DOI:** 10.3389/fphar.2022.820210

**Published:** 2022-02-14

**Authors:** Dasiel O. Borroto-Escuela, Ramón Fores, Mariana Pita, Miguel A. Barbancho, Pablo Zamorano‐Gonzalez, Natalia García Casares, Kjell Fuxe, Manuel Narváez

**Affiliations:** ^1^ Department of Neuroscience, Karolinska Institute, Stockholm, Sweden; ^2^ Department of Biomolecular Science, Section of Physiology, University of Urbino, Urbino, Italy; ^3^ Grupo Bohío-Estudio, Observatorio Cubano de Neurociencias, Yaguajay, Cuba; ^4^ Instituto de Investigación Biomédica de Málaga, Facultad de Medicina, Universidad de Málaga, Málaga, Spain; ^5^ Departamento de Neurogenética, Instituto de Neurología y Neurocirugía, La Habana, Cuba

**Keywords:** galanin receptor 2, neuropeptide Y receptor 1, spatial memory, neurogenesis, receptor-receptor heterodimers

## Abstract

A need for new therapeutic approaches are necessary for dementia conditions and memory deficits of different origins, such as Alzheimer's disease. There is complex pathophysiological mechanisms involved, affecting adult hippocampal neurogenesis, in which neuropeptides and its neurogenesis regulation seem to participate. Neuropeptide Y(NPY) Y1 receptor (Y1R) and galanin (GAL) receptor 2 (GALR2) interact in brain regions responsible for learning and memory processes, emphasizing the hippocampus. Moreover, a significant challenge for treatments involving peptide drugs is bypassing the blood-brain barrier. The current study assesses the sustained memory performance induced by GALR2 and NPYY1R agonists intranasal coadministration and their neurochemical hippocampal correlates. Memory retrieval was conducted in the object-in-place task together with in situ proximity ligation assay (PLA) to manifest the formation of GALR2/Y1R heteroreceptor complexes and their dynamics under the different treatments. We evaluated cell proliferation through a 5-Bromo-2’-deoxyuridine (BrdU) expression study within the dentate gyrus of the dorsal hippocampus. The GalR2 agonist M1145 was demonstrated to act with the Y1R agonist to improve memory retrieval at 24 hours in the object-in-place task. Our data show that the intranasal administration is a feasible technique for directly delivering Galanin or Neuropeptide Y compounds into CNS. Moreover, we observed the ability of the co-agonist treatment to enhance the cell proliferation in the DG of the dorsal hippocampus through 5- Bromo-2’-deoxyuridine (BrdU) expression analysis at 24 hours. The understanding of the cellular mechanisms was achieved by analyzing the GALR2/Y1R heteroreceptor complexes upon agonist coactivation of their two types of receptor protomers in Doublecortin-expressing neuroblasts. Our results may provide the basis for developing heterobivalent agonist pharmacophores, targeting GALR2-Y1R heterocomplexes. It involves especially the neuronal precursor cells of the dentate gyrus in the dorsal hippocampus for the novel treatment of neurodegenerative pathologies as in the Alzheimer’s disease.

## 1 Introduction

Declarative memory is a procedure for recalling facts and events where the posterior hippocampus (dorsal, in rodents) achieves integration and consolidation. Moreover, by connecting spatial and non-spatial data, this hippocampal region determines the contextual component of declarative processes (Fanselow and Dong, 2010; Aimone et al., 2014; Baptista and Andrade, 2018).

Hippocampal-dependent memory and learning are related to the process of neurogenesis by incorporating young cells into this dorsal hippocampal circuit. (Wu and Hen, 2014). Eriksson et al. (1998) primary confirmed the presence of neurogenesis in humans. Their results were reinforced (Spalding et al., 2013; Boldrini et al., 2018; Moreno-Jimenez et al., 2019) and doubted (Cipriani et al., 2018; Sorrells et al., 2018) by further reports. However, this discrepancy was due to the divergence of tissue processing procedures and the limited obtainability of correctly preserved human brain tissue samples (Kempermann et al., 2018).

Adult hippocampal neurogenesis emerges during physiological ageing in humans as a robust phenomenon to keep healthy cognitive functions. In this view, previous data support the persistence of adult neurogenesis until the ninth decade of life (Moreno-Jimenez et al., 2019). Infants and older people have higher and lower rates of neuron formation; thus, a key influence on neurogenesis is the age itself (Spalding et al., 2013). In fact, the ageing population is becoming more usual clinical problems as dementia states and neurodegenerative memory deficits such as Alzheimer’s disease (AD). In AD, the expansion of misfolded proteins reduces gradually neurogenesis leading to memory loss and cognitive deterioration (Kempermann et al., 2015). Consequently, the pathophysiology of AD is related to hippocampal neurogenesis impairment displaying a significant cause of dementia (Llorens-Martin et al., 2013). Accordingly, AD has neuropathological mechanisms damaging immature neurons, distant from physiological ageing.

Currently, the existing pharmacological cognitive enhancers (Memantine, Galantamine, Donepezil, rivastigmine) do not improve the AD progression, and their actions are only symptomatic and transient (Borbely et al., 2013). There is an incipient requirement to clarify AD multifaceted pathophysiological mechanisms and novel goals and recognize crucial mediators for future drug advances. Neuropeptides are extensively spread in brain regions involved in memory and learning procedures, emphasizing the hippocampus and neurogenesis regulation (Zaben and Gray, 2013).

The widely distributed CNS, Neuropeptide Y (NPY), regulates many diverse physiological purposes such as stress response, anxiety, and cognition (Enman et al., 2015; Reichmann and Holzer, 2016). Depending on receptor subtype stimulated in discrete brain regions and the kind of memory studied, NPY shows both stimulatory and inhibitory outcomes in memory and learning. In the aged rat hippocampus, the decreased NPY expression was correlated to memory diminishing and ablation of neurogenesis (Borbely et al., 2013). Similarly, in AD patients were shown reduced both NPY receptor densities in hippocampal and cortical regions (Martel et al., 1990) and NPY levels in plasma and cerebrospinal fluid samples (Nilsson et al., 2001). A pivotal role in memory and spatial learning is associated with Y1 receptors (Y1R) in the hippocampus (Sperk et al., 2007). An upregulated NPY mRNA expression levels in the hippocampal dentate gyrus (DG) were shown after spatial learning tasks in rats (Hadad-Ophir et al., 2014). Moreover, in rats with an AD-like phenotype, the intracerebroventricular injection of NPY or Y1R agonist improves spatial memory deficits (Rangani et al., 2012). Furthermore, *in vivo* injection of NPY induced neurogenesis in the DG of the hippocampus through Y1R (Decressac et al., 2011; Geloso et al., 2015).

Galanin (GAL) is an additional neuropeptide that is broadly distributed in the brain of mammals (Tatemoto et al., 1983). Central GAL is related to several physiological roles, including effects on memory and learning *via* classical neurotransmitters modulation (Lang et al., 2015). Furthermore, GAL is implicated in psychiatric and neurological disorders, such as AD (Counts et al., 2010). Previous evidence indicated a negative correlation of neurodegenerative signs and cognitive impairment in AD patients related to increased GAL autoantibody levels in their cerebrospinal fluid (Costa et al., 2011). Furthermore, data demonstrated that the GAL2 receptors (GALR2) are responsible for the favourable features of GAL on memory-improving and hippocampal toxicity in a rat model of AD (Li et al., 2013). Depending on the dose or administration site, learning improvement, an inhibitory effect or lack of outcome have also been observed after GAL administration (Beck and Pourie, 2013). Regarding neurogenesis, the GalR2/3 agonist, Galanin 2–11, induced proliferation of hippocampal precursor cells and seems to produce granule cell neurons of the dentate gyrus (Abbosh et al., 2011).

We have defined numerous GAL and Y1R interactions in diverse limbic system regions, with behavioural, region-specific, cellular, and molecular connections (Narvaez et al., 2015; Narvaez et al., 2016; Narvaez et al., 2018). Recently, a facilitatory GAL/Y1R interaction was exposed, concerning the formation of GALR2/Y1R heteroreceptor complexes in the dentate gyrus of the ventral hippocampus. Moreover, GALR2 stimulation enhanced Y1R agonist-mediated antidepressant activities in the forced swimming test (Borroto-Escuela et al., 2021).

Bypassing the blood-brain barrier is a considerable challenge for significant size peptides. Consequently, intranasal (i.n.) administration is a feasible strategy for the direct delivery of protein therapeutics rapidly into the CNS, supported by preclinical and clinical experiments (Lochhead and Thorne, 2012). This administration has several benefits, such as fewer side effects than peripheral administration and the comfort of non-invasiveness application (Chapman et al., 2013). Emerging evidence indicates that i.n. direct delivery of NPY (Nahvi et al., 2021), Y1R agonists (Serova et al., 2017) or GALR2 agonists (Yun et al., 2019) to the brain might have therapeutic potential.

In the present study, we assessed the beneficial actions of i.n. coadministered GALR2 and Y1R agonists on sustained memory performance and their neurochemical hippocampal correlates. Memory retrieval was conducted in the object-in-place task in common with the *in situ* proximity ligation assay (PLA) to label GALR2/Y1R heteroreceptor complexes establishment and their dynamics under the diverse administrations. We assessed cell proliferation through a 5-Bromo-2′-deoxyuridine (BrdU) expression analysis within the dentate gyrus of the dorsal hippocampus.

## 2 Materials and Methods

### 2.1 Animals

Male Sprague-Dawley rats from CRIFFA (Barcelona; 200-250gr; 6–8 weeks) had ad-libitum food and water access. They were preserved under the standard 12 h dark/light cycle, with controlled relative humidity (55–60%) and temperature (22 ± 2°C). Guidelines for preclinical experiments were performed following EU Directive 2010/63/EU and Spanish Directive (Real Decretory 53/2013) approvals. All dealings concerned with an experimental treatment, housing and maintenance of the rats were permitted by the Local Animal Ethics, Care, and Use Committee for the University of Málaga, Spain.

### 2.2 Drugs Used

Diluted peptides were recently prepared in distilled water. Galanin receptor 2 agonist (M1145), Y1R receptor agonist [Leu^31^, Pro^34^] NPY, GALR2 Antagonist M871 were acquired from Tocris Bioscience (Bristol, United Kingdom). A detailed report is accessible in Supplement material on intranasal (i.n.) delivery of peptides.

### 2.3 Behavioural Testing

#### 2.3.1 Object-in-Place Task

**FIGURE 1:**
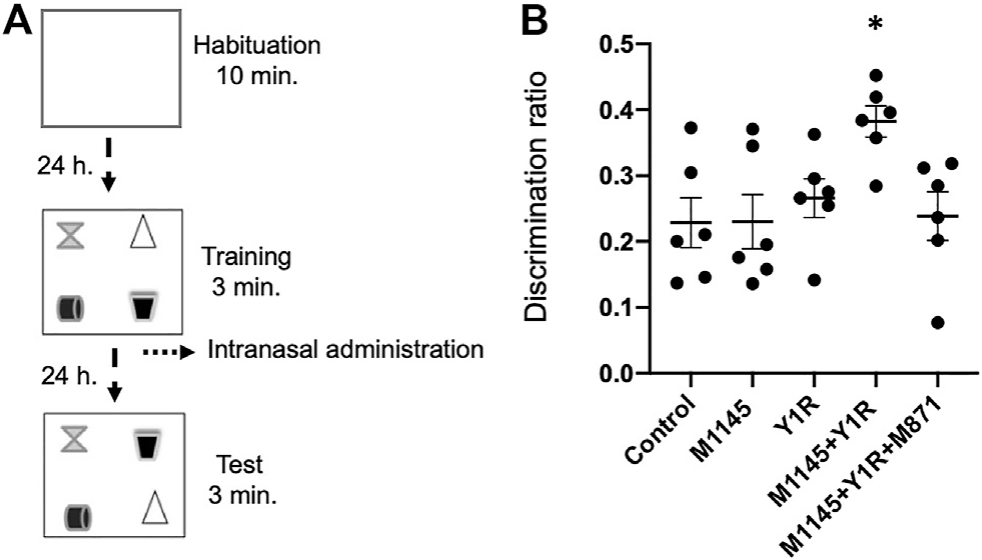
Behavioural effects produced by Galanin 2 receptor agonist (M1145) and the Neuropeptide Y Y1 receptor (NPY Y1) agonist alone and in combination on retrieval in the Object-in-Place memory task. **(A)** Schematic representation of the procedures followed to perform the behavioural experiments in the Object-in-Place task. The animals performed the task in three phases, separated 24 h from each other, where they explored freely during 10 min in the habituation phase without objects, 3 min in the training phase with four different objects, and finally 3 min in the test phase with two of the objects with the exchanged position. The pharmacological treatments were administered intranasally to the different groups of animals 24 h before the testing phase. **(B)** The graphic shows the ability of rats to discriminate the exchanged objects at 24 h post-training after the administration of M1145 in combination with the NPY Y1 agonist in the Object-in-Place task. The response induced by M1145 and the NPYY1 coadministration after the acquisition phase improves object-in-place performance following a 24-h delay. Moreover, this effect is blocked by the GAL 2 receptor (GALR2) antagonist M871. Data represent mean ± SEM of the discrimination ratio during the 3 min of the test phase. N = 6 animals in each group. *p < 0.05 vs. the rest of the groups according to one-way ANOVA (F4, 25 = 3.56) followed by Newman-Keuls post-hoc test. Abbreviations: Control = Distilled water; M1145 = Galanin 2 receptor agonist 132 µg; Y1R = Y1R receptor agonist [Leu^31^-Pro^34^]NPY 132 µg; M1145 + Y1R = Coadministration of M1145 and Y1R; M1145 + Y1R + M871 = Coadministration of M1145, Y1R and GALR2 antagonist M871 132 µg.

The object-in-Place task was developed to evaluate memory based upon spontaneous object exploration behaviours (Warburton and Brown, 2015). The object-in-Place task evaluates spatial hippocampal memory and is less aversive than the Morris water maze task. Each rat was exposed to the task to assess memory retrieval at 24 h using a plastic open field 100 × 100 × 60 cm (length × width × height) under dim light. During the behavioural period, the rats were single-housed. The task trials contain three phases: habituation, training, and test (Ampuero et al., 2013; Barker and Warburton, 2015) (Figure 1A):

#### 2.3.2 Habituation

Animals were handled for 2 days, then familiarized to the empty arena for 10 min (1 trial, 10 min).

#### 2.3.3 Training

24 h later, in the training phase, each animal was placed in the centre of the arena, which contained four distinct objects and allowed to investigate them (1 trial, 3 min). The objects varied in shape and colour with similar weight and size, placed in the corners 10 cm from the sidewall and could not be displaced. After the exploration, all objects were cleaned with 5% ethanol.

#### 2.3.4 Test

The test session was performed 24 h post-training, in which two of the objects had exchanged location and animals were allowed to examine the objects (1 trial, 3 min). Exploration was described as time spent sniffing or touching the object with the nose or forepaws. The discrimination capacity was represented by the time spent investigating the objects in the changed position (C) compared with the time spent exploring the objects in the same place (S). A discrimination ratio was calculated as DI = (C–S)/(C + S). Integral object-in-place memory happens when the animal employs more time examining the 2 objects in different locations than the same ones. An overhead video camera monitored and recorded the animal’s behaviour, which was scored and analyzed blind to the treatment, using the Raton Time 1.0 software (Fixma S.L., Valencia, Spain). We also examined the locomotor activity using the video-tracking software EthovisionXT (Noldus, Wageningen, Nederland). Between trials, object position was counterbalanced between rats and the arena and the objects were carefully cleaned with 5% ethanol. The intranasal treatments were administered 24 h before the test phase. In addition, the total exploration time (Table 1) and the locomotor activity (Table 2) between the experimental groups were controlled to demonstrate that treatment did not affect the exploration ability of the rats.

**TABLE 1:** Exploratory activity of rats treated with Galanin 2 receptor agonist (M1145) and the Neuropeptide Y Y1 receptor (NPY Y1) agonist alone and in combination. Data are expressed as mean ± SEM from the training and test sessions during the Object-in-place task. For total exploration time in training and test sessions no statistically significant difference was observed between the experimental groups according to one way ANOVA. && *p* < 0.01 according to one-way ANOVA followed by Newman-Keuls post-hoc test for relocated objects time of M1145 + Y1R group compared with the rest of the groups. ***p < 0.001 (two-tailed paired *t*-test) Familiar vs. Relocated. Abbreviations: Control = Distilled water; M1145 = Galanin 2 receptor agonist 132 µg; Y1R = Y1R receptor agonist [Leu^31^-Pro^34^]NPY 132 µg; M1145 + Y1R = Coadministration of M1145 and Y1R; M1145 + Y1R + M871 = Coadministration of M1145, Y1R and GALR2 antagonist M871 132 µg.

Group	Control	M1145	Y1R	M1145 + Y1R	M1145 + Y1R + M871
Training [Total exploration time (s)]	20.6 ± 0.8	21 ± 0.4	21.4 ± 0.8	21 ± 0.6	22 ± 1.1
Test [Total exploration time (s)]	19.8 ± 1.6	18.2 ± 1.1	20.1 ± 0.9	22.3 ± 1.2	19.3 ± 0.9
Familiar Objects (s)	7.7 ± 0.8	7.1 ± 0.7	7.4 ± 0.5	6.9 ± 0.5	7.4 ± 0.5
Relocated objects (s)	12.1 ± 0.8***	11 ± 0.4***	12.7 ± 0.6***	15.4 ± 0.8***&&	11.9 ± 0.7***

**TABLE 2:** Locomotor/spontaneous activity in the Object-in-place task. Data are expressed as mean ± SEM. According to one-way ANOVA, no statistically significant difference was observed between the experimental groups. Abbreviations: Control = Distilled water; M1145 = Galanin 2 receptor agonist 132 µg; Y1R = Y1R receptor agonist [Leu^31^-Pro^34^]NPY 132 µg; M1145 + Y1R = Coadministration of M1145 and Y1R; M1145 + Y1R + M871 = Coadministration of M1145, Y1R and GALR2 antagonist M871 132 µg.

Group	Control	M1145	Y1R	M1145 + Y1R	M1145 + Y1R + M871
Locomotor Activity (cm)	2080 ± 118	2227 ± 124	2048 ± 142	2181 ± 127	2140 ± 113

### 2.4 Evaluation of Hippocampal Cell Proliferation

For examination of BrdU-positive cells, a distinctive set of rats was administered two injections of 5′ -Bromo-2′ -deoxyuridine (BrdU, cat. no. B5002, Sigma, St. Louis, MO, United States) dissolved at 15 mg/ml in a sterile 0.9% NaCl solution. BrdU was injected intraperitoneally (i.p.) during the ad libitum feeding period at 50 mg/kg body weight dose (every 2 h after the i.n. treatments, starting at 9:00 a.m.). Twenty-4 hours after the i.n. administration, rats were profoundly anaesthetized with pentobarbital (Mebumal; 100 mg/kg, i.p.) and transcardially perfused with 4% paraformaldehyde (wt/vol, Sigma). Brains were coronally sliced (30 μm-thick) through the dorsal hippocampus (posterior in primates) (−1.60 to −5.30 Bregma; Paxinos and Watson, 1986).

Animals were allocated arbitrarily into five experimental groups: 1) Control: distilled water; 2) M1145- treated group (132 µg); 3) Y1R-treated group receiving the Y1R agonist [Leu^31^, Pro^34^] NPY (132 µg); 4) M1145 + Y1R: group administered with both substances; 5) M1145 + Y1R + M871: group treated with M1145, [Leu^31^, Pro^34^] NPY and the GALR2 antagonist (M871; 132 µg) (N = 4 in each group). The BrdU procedure (Pilar-Cuellar et al., 2012; Abrial et al., 2014; Borroto-Escuela et al., 2021) is based on previously published procedures.

### 2.5 Immunohistochemistry

Brain sections were incubated free-floating in saline sodium citrate buffer (pH 6; 10 nM sodium citrate) for 90 min at 65°C, followed by 30 min with 0.6% H2O2 to remove endogenous peroxidases. After 30 min in 2 M hydrochloric acid (HCl) to denature deoxyribonucleic acid (DNA), sections were incubated for neutralization with 0.1 M sodium borate (pH 8). Then, slices were incubated at 4°C overnight with a primary antibody against BrdU (1:1,000, Abcam, United States) in 2.5% donkey serum. Following additional washed with PBS and incubated with a secondary antibody for 90 min (biotinylated anti-rabbit IgG, 1/200, Vector Laboratories), sections were amplified with ExtrAvidin peroxidase (Sigma, St. Louis, MO, United States) diluted 1:1,000 in darkness at room temperature for 1 hour. Immunolabeling was exposed with 0.05% diaminobenzidine (DAB; Sigma) and 0.03% H2O2 in PBS. After various washes, sections were mounted on gelatin-coated slides, dehydrated in graded alcohols, and cover-slipped in DePeX mounting medium (VWR). As previously described, BrdU-labelled cells were analyzed using the optical fractionator method in unbiased stereological microscopy (Olympus BX51 Microscope, Olympus, Denmark) (see Supplement Material for details).

### 2.6 *In situ* Proximity Ligation Assay

The *in situ* proximity ligation assay (*in situ* PLA) was performed as described previously (Borroto-Escuela et al., 2013; Narváez et al., 2021) to report the GALR2-Y1R heteroreceptor complexes. Treated rats were distributed into experimental groups: 1) Control: distilled water; 2) M1145- treated group (132 µg); 3) Y1R-treated group receiving an Y1R agonist [Leu^31^, Pro^34^]NPY (132 µg); 4) M1145 + Y1R: group administered with both substances; 5) M1145 + Y1R + M871: group treated with M1145, [Leu^31^, Pro^34^]NPY and the GALR2 antagonist (M871; 132 µg) (*n* = 4 in each group). Animals were perfused with 4% paraformaldehyde 24 h after i.n. administration, brains were removed, and sections were prepared.

Slices were free-floating, washed four times with PBS and quenched with 10 mM Glycine buffer for 20 min at room temperature. Subsequently, sections were permeabilized [Fetal bovine serum (FBS) 10% and Triton X- 100, 0.5%, in Tris buffer saline (TBS), pH 7.4] during 30 min at room temperature. Slices were incubated with the blocking buffer [0.2% bovine serum albumin (BSA) in PBS] for 30 min at room temperature after washes. A new incubation was achieved with the primary antibodies (GALR2 rabbit, Alomone Lab, 1:100; Y1R goat, sc-21992 Santa Cruz Biotechnology INC, CA, 1:200) diluted in the blocking solution at 4°C overnight. Afterwards, the proximity probe mixture (Duolink PLA probe anti-mouse MINUS and Duolink PLA probe anti-rabbit PLUS, Sigma-Aldrich, Stockholm, Sweden) was applied to the slices and incubated for 1 hour 37°C. After washing, the sections were incubated with the hybridization-ligation solution (BSA, 250 g/ml), T4 DNA ligase (final concentration of 0.05 U/μl), 0.05% Tween-20, 250 mM NaCl, 1 mM ATP, and the circularization or connector oligonucleotides (125–250 nM)) at 37°C for 30 min. The rolling circle amplification mixture (Duolink amplification red, DUO82011, Sigma-Aldrich, Stockholm, Sweden) was incubated at 37°C for 100 min. Then, the sections were incubated with the uncovering solution at 37°C for 30 min. The free-floating slices were put on a microscope slide with mounting medium (Duolink Mounting Medium, Sigma-Aldrich) containing (4′,6-diamidino-2-phenylindole) DAPI, a nuclear blue counterstain. Negative control was performed, excluding the species-specific primary antibody corresponding to the GALR2 in the presence of the two PLA probes. For positive control, a parallel analysis of the 5-HTR1A-5HTR2A isoreceptor complexes was completed as previously published (Borroto-Escuela et al., 2017). *In situ* PLA image achievement and data evaluation were made as previously described (Narvaez et al., 2020; Borroto-Escuela et al., 2021).

### 2.7 Statistical Analysis

Data are offered as mean ± SEM, and sample number (*n*) is specified in figure legends. All data were analyzed using GraphPad PRISM 8.0 (GraphPad Software, La Jolla, CA, United States). One-way analysis of variance (ANOVA) followed by the Newman-Keuls comparison post-test was performed. Paired Student’s t-tests (two-tailed) were used to examine whether individual groups had discriminated between the objects in the object-in-place task. Differences were considered significant at *p* < 0.05 (*p < 0.05 **p < 0.01 ***p < 0.001).

## 3 Results

### 3.1 GALR2 Agonist and Y1R Agonist Intranasally-Administered Interaction Enhanced Memory Retrieval in the Object-in-Place Task

In the Object-in-Place task, rats explore freely for 10 min in the habituation phase without objects and 3 min in the training phase with four different objects. Finally, 24 hours after the intranasal (i.n.) administration was performed, the test phase was 3 min with two objects with exchanged positions to assess drug effects on retrieval of object-in-place memory (Figure 1A).

The administration of the GalR2 agonist M1145 alone and the Y1R agonist alone lacked effects on the object-in-place memory task (Figure 1B) compared with the control group. However, upon the M1145 and the Y1R i.n. coadministration after the acquisition phase, improved object-in-place memory retrieval was observed following a 24-h delay (one-way ANOVA, F4,25 = 3.56, *p* < 0.05, Newman-Keuls post-hoc test: *p* < 0.05; Figure 1B) compared with the rest of the groups. GALR2 involvement in this outcome was certified since the addition of the GALR2 antagonist M871 neutralized enhancing memory retrieval (Newman-Keuls post-hoc test: *p* < 0.05; Figure 1B) induced by the coadministration of M1145 and Y1R agonist in the object-in-place task.

Furthermore, the training and test sessions’ total exploration time was calculated. Data indicated that the exploration ability of rats was not affected by the treatments (Table 1). Within-group analyses showed that Control (*t* = 8.22; df = 5; *p* < 0.001), M1145 (*t* = 7.90; df = 5; *p* < 0.001), Y1R (*t* = 8.70; df = 5; *p* < 0.001), M1145 + Y1R (*t* = 13.39; df = 5; *p* < 0.001) and M1145 + Y1R + M871 (*t* = 6,105; df = 5; *p* < 0.001) groups showed a significant preference for the two objects that had changed position compared with objects that had remained in the same position. Moreover, the time exploring the relocated object was significantly higher (one-way ANOVA, F4,25 = 6.15, *p* < 0.01, Newman-Keuls post-hoc test: *p* < 0.01) in the M1145 + Y1R group compared with the rest of the groups (Table 1). Spontaneous motor behaviour showed no treatment effects (see data of the locomotor activity in Table 2).

### 3.2 GALR2 Agonist and Y1R Agonist Intranasal Coadministration Increased Cell Proliferation in the Dorsal Hippocampus

**FIGURE 2:**
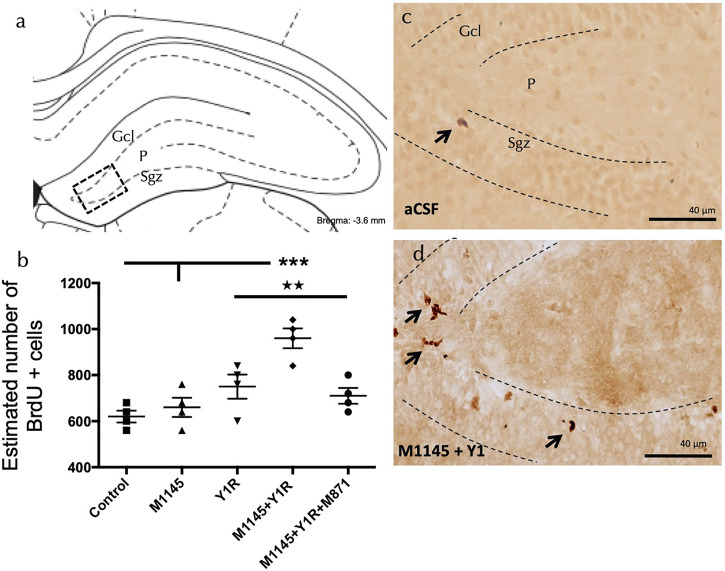
5′-Bromo-2′-deoxyuridine (BrdU) immunolabelling (BrdU+) in the dentate gyrus of the dorsal hippocampus, after the intranasal (in) administration of Galanin 2 receptor agonist (M1145) and Y1R receptor agonist, either alone or in combination with or without the GAL 2 receptor antagonist (M871). **(A,D)** The majority of the BrdU-labeled cells were located in the subgranular zone (Sgz) of the dentate gyrus at the border between the granular cell layer (Gcl) and the polymorphic layer (P) of the dentate gyrus in the dorsal hippocampus. They appeared to be nerve cells forming clusters of 3–4 cells. (Bregma: -3.6 mm; according to the Paxinos and Watson stereotaxic atlas (1986)). **(B)** Quantification of total BrdU IR cells in the dentate gyrus of the dorsal hippocampus. Data represent mean ± SEM to show the differences between groups after administration of aCSF, M1145, Y1R agonist [Leu^31^-Pro^34^]NPY, or the coadministration of both peptides with or without M871. M1145 and the Y1R agonist coadministration increased the number of cells with BrdU expression in the dorsal hippocampus compared to the lack of effects of the two peptides given alone and the aCSF group. Furthermore, this effect was blocked by GALR2 antagonist M871. ***p < 0.001 vs. control and M1145; ★★ *p* < 0.01 vs. Y1R and M1145 + Y1R + M871 according to one-way ANOVA (F4, 15 = 10.96, *p* < 0.001) followed by Newman-Keuls post-hoc test. Inter-group comparisons are indicated by the vertical lines from the horizontal line above bars. N = 4 in each group. M1145 and Y1R agonist coinjection **(D)** increased the BrdU immunolabelling in Sgz in the dentate gyrus compared with the control group **(C)**. Arrows indicate examples of clusters of BrdU positive nerve cells. Dashed lines outline the Gcl of the dentate gyrus. Abbreviations: Control = Distilled water; M1145 = Galanin 2 receptor agonist 132 µg; Y1R = Y1R receptor agonist [Leu^31^-Pro^34^]NPY 132 µg; M1145 + Y1R = Coadministration of M1145 and Y1R; M1145 + Y1R + M871 = Co-administration of M1145, Y1R and GALR2 antagonist M871 132 µg. Treatments were performed 24 h before brain processing; see material and methods for further details.

To investigate the cellular mechanisms associated with the behavioural effects, we assessed the impact of GALR2 and Y1R agonist intranasal (i.n.) coadministration on adult dorsal hippocampal cell proliferation based on using the thymidine analogue 5-Bromo-2′-deoxyuridine (BrdU) (Figure 2A–D).

The i.n. administration of the Y1R agonist induced no significant changes in the number of BrdU-IR profiles in the subgranular zone (Sgz) of the dentate gyrus (Figure 2B) compared with the control group. The administration of GalR2 agonist M1145 alone lacked effects on the numbers of BrdU-IR profiles (Figure 2B) compared with the control group (Figures 2B,C).

Conversely, M114545 and Y1R agonist coinjection significantly increased the number of BrdU-IR profiles, specifically in the Sgz of the dentate gyrus compared with the M1145 and the control groups (one-way ANOVA, F4, 15 = 10.70, *p* < 0.001, Newman-Keuls post-hoc test: *p* < 0.001) and with the Y1R agonist alone group (Newman-Keuls post-hoc test: *p* < 0.01) (Figures 2B,D). The cotreatment with the GALR2 antagonist M871 completely blocked the M1145 and Y1R agonists action in the dentate gyrus (Newman-Keuls post-hoc test: *p* < 0.01) (Figure 2B), indicating the participation of GALR2 in the M1145/NPYY1R agonist interaction to stimulate cell proliferation.

### 3.3 Agonist Coactivation of GALR2 and Y1R Increases GALR2/Y1R Heteroreceptor Complexes Within the Dentate Gyrus of the Dorsal Hippocampus. Association to Doublecortin-Expressing Cells

To examine the cellular mechanism associated with the detected effects on cell proliferation, we performed *in situ* proximity ligation assay (PLA) on the dorsal hippocampal dentate gyrus (DG) of the dorsal hippocampus, studying the GALR2-Y1R heterocomplexes after GALR2 and Y1R agonists administration.

**FIGURE 3:**
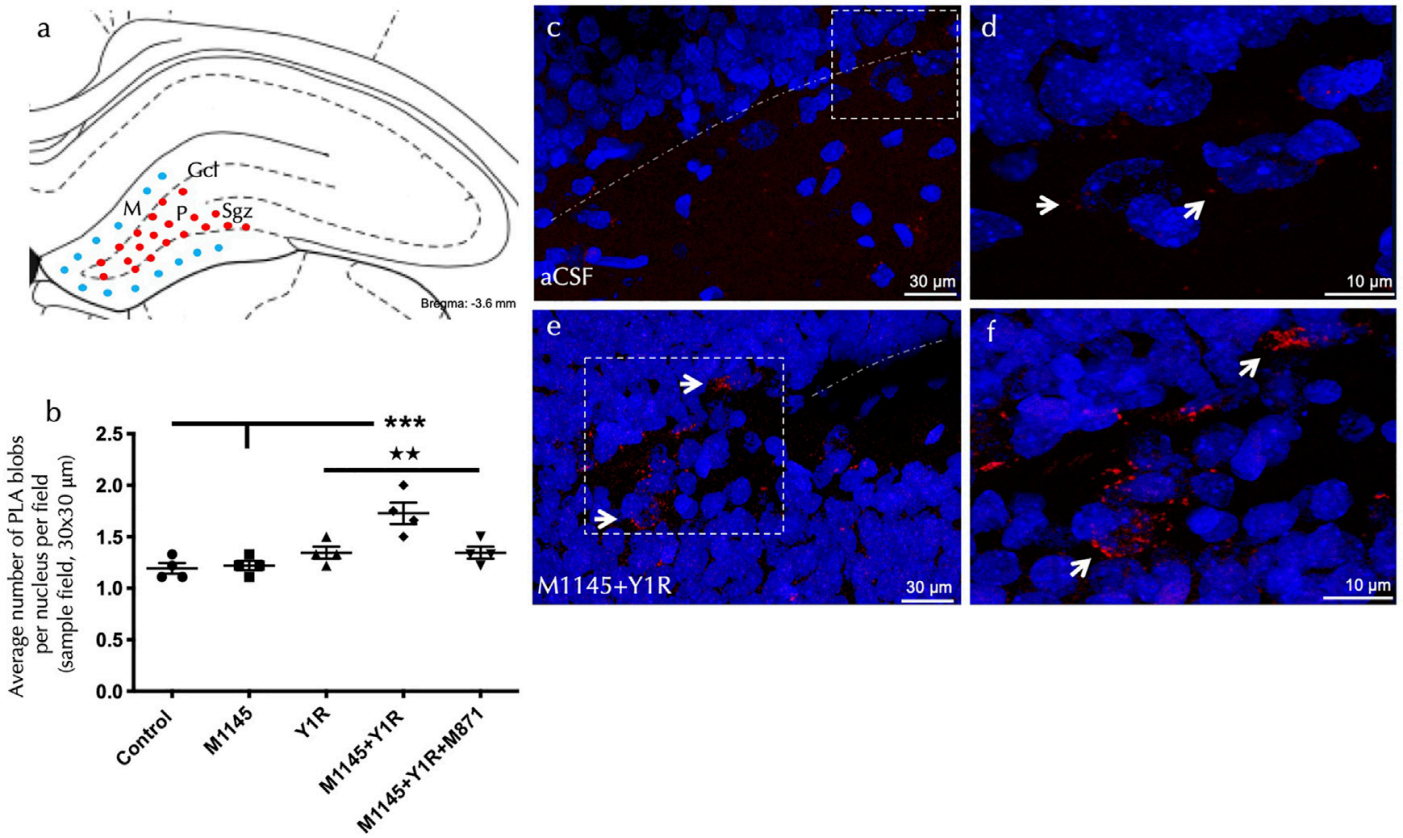
GALR2/Y1R heteroreceptor complexes are detected by *in situ* PLA in the dorsal dentate gyrus (DG). **(A)** The diagram shows the presence of positive red PLA signals (red circles) mainly in the subgranular zone (Sgz) of the dentate gyrus at the border between the granular cell layer (Gcl) and polymorphic layer (P) of the dentate gyrus in the dorsal hippocampus. PLA positive red signals were also observed in the polymorphic layer. Blue-filled circles indicate a negative PLA signal in the molecular layer (M). [Bregma: –3.6 mm; according to the Paxinos and Watson (1998) stereotaxic atlas]. **(B)** Quantification of PLA signals in Sgz was performed by measuring red PLA positive blobs per nucleus per sampled field by an experimenter blind to treatment conditions. Sprague Dawley rats significantly increased GALR2-Y1R heterocomplexes (PLA blobs) in the Sgz after M1145 and Y1R agonist intranasal administration. This effect was blocked by treatment with the GALR2 antagonist M871. ***p < 0.001 vs. control and M1145; ★★ *p* < 0.01 vs. Y1R and M1145 + Y1R + M871 according to one-way ANOVA (F4, 15 = 10.21, *p* < 0.001) followed by Newman-Keuls post-hoc test. Inter-group comparisons are indicated by the vertical lines from the horizontal line above bars. Data are expressed as mean ± SEM, four rats per group, duplicates). **(C–F)** Representative microphotographs of the significant increase in the density of GALR2-Y1R positive red PLA blobs in the Sgz sub-region after GAL and Y1R agonist coinjection **(E)** compared with the control group **(C)**. Magnified views from dashed boxes in **(C,E)** are shown in **(D,F)**, respectively. GALR2-Y1R heteroreceptor complexes are shown as red PLA blobs (clusters) found in high densities per cell in many nerve cells using confocal laser microscopy. White arrows point to PLA positive clusters. Dashed lines outline the Gcl of the dentate gyrus. The nuclei are shown in blue by DAPI. Abbreviations: Control = Distilled water; M1145 = Galanin 2 receptor agonist 132 µg; Y1R = Y1R receptor agonist [Leu^31^-Pro^34^]NPY 132 µg; M1145 + Y1R = Coadministration of M1145 and Y1R; M1145 + Y1R + M871 = Coadministration of M1145, Y1R and GALR2 antagonist M871 132 µg. Treatments were performed 24 h before brain processing; see material and methods for further details.

PLA-positive red clusters were explicitly located in the subgranular zone and the polymorphic layer of the dorsal DG (Figure 3A).

Quantification of PLA density confirmed an increase in the density of the PLA-positive red clusters after the co-administration of M1145 and YR1 agonist compared to control (one-way ANOVA, F4, 15 = 10.21, *p* < 0.001, Newman-Keuls post-hoc test: *p* < 0.001); M1145 group (Newman-Keuls post-hoc test: *p* < 0.001) or Y1R agonist alone (Newman-Keuls post-hoc test: *p* < 0.01) (Figures 3B–F). Similarly to the BrdU-IR profiles response described above, the i.n. administration of the Y1R agonist induced no significant increase in the density of the PLA-positive red clusters (Figure 3B). The administration of M1145 alone lacked effects on the PLA-positive red clusters compared with the control group (Figure 3B). Again, the presence of the GALR2 antagonist M871 entirely blocked this increase (Newman-Keuls post-hoc test: *p* < 0.01) (Figure 3B), demonstrating the contribution of GALR2 in this interaction. The signal specificity was certified with an absence of PLA clusters observed in the molecular layer of the DG, an area that appears to lack GALR2 (O’donnell et al., 1999).

Furthermore, to explore the specific cell population possibly involved in the abovementioned results, we analyzed the neuroblast-expressing marker Doublecortin (Supplementary Figure S1). We found a specific colocalization of GALR2 (Supplementary Figure S1A) and Y1R (Supplementary Figure S1B) with Doublecortin-expressing cells in the dorsal dentate gyrus. We propose a specific role for allosteric GALR2-Y1R interactions in the neuroblasts located in the DG of the dorsal hippocampus, while GALR1 did not colocalize with Doublecortin-expressing cells.

Taken together, GALR2 and Y1R coactivation may allow the formation of GALR2-Y1R heterocomplexes in neuroblastic cells in the dorsal dentate gyrus, increasing cellular proliferation and their participation in the spatial-learning effects observed.

## 4 Discussion

For the primary instance, the current work proves the existence of GALR2-Y1R hetero-complexes in the dentate gyrus (DG) of the dorsal hippocampus, principally found in the subgranular zone, upon combined GALR2- and Y1R agonist intranasal treatment. The GalR2 agonist M1145 was demonstrated to act with the Y1R agonist to improve memory retrieval at 24 h in the object-in-place task, which strongly supports the enhancement of spatial learning. The mechanism likely involves their positive allosteric receptor-receptor interactions in these complexes. In humans, the hippocampus is a critical brain region for object-in-place memory formation (Owen et al., 1995; Milner et al., 1997). Moreover, patients with Alzheimer’s Disease show disturbed object-in-place associative recognition memory (Fowler et al., 2002). The hippocampus also has a patent role in object recognition memory tasks with a spatial component, such as the object-in-place task (Barker and Warburton, 2011a). Consequently, hippocampal lesions impair object-in-place tasks (Mumby et al., 2002; Barker and Warburton, 2011b) and electrophysiological reports verified that spatial location of objects is detected by hippocampal neurons (Muller, 1996). Moreover, selective inactivation of the dentate gyrus (DG) subregion of the hippocampus has been shown to impair the encoding of object location memory (Barbosa et al., 2012).

An area of debate among researchers who use spontaneous recognition tasks is to consider if the innate bias in animals’ exploratory behaviour towards the new (or relocated) object/s reflects or not underlying memory discrimination or sensitivity (Ennaceur, 2010). Recently, this concern seems to be solved since is provided empirical validation of the Discrimination Ratio as a measure of recognition memory sensitivity in humans, not bias (Sivakumaran et al., 2018). Furthermore, the authors suggest that animal researchers should be confident in interpreting the Discrimination Ratio as an analogue of recognition memory sensitivity. These results validate both the within-group analyses to detect the preference for the new object (or relocated) or not, and the inter-group comparisons for the level of discrimination (Ennaceur et al., 1997). We observed a preference for the relocated objects in all the groups, while a specific increase in the discrimination ratio after the subthreshold doses of M1145 or the Y1R agonist intranasal coadministration. These results would reflect a memory improvement in the object-in-place task after the coadministration of both agonists.

Intranasal administration is a suitable technique for directly carrying protein therapeutics to the CNS, successfully bypassing the blood-brain barrier and getting the brain by two potential pathways. The first route implicates the drug reaching the submucosa throughout the nasal epithelium, after which the preparation might get straight access to the CSF or enter the CNS using volume transmission across extracellular diffusion within perineuronal channels (Fuxe et al., 2010; Borroto-Escuela et al., 2015). The second route can involve incorporating the drug into primary olfactory neurons, intracellularly transported to the olfactory bulb and delivered to additional brain regions through volume transmission and neuronal intracellular. The transference using 125-I–labelled combinations given intranasally into the brain was more significant than achieved under intravenous administration (Thorne and Frey II, 2001). Consequently, access into the brain of insulin, leptin, melatonin, oxytocin, angiotensin, arginine-vasopressin, IGF-1, Glucagon-like peptide-2, rhNGF and vasointestinal polypeptide has been demonstrated using i.n. pathway (Chapman et al., 2013; Sasaki-Hamada et al., 2017; Striepens et al., 2013). Emergent data indicate that NPY intranasal distribution to the brain has therapeutic potential (Serova et al., 2020; Nahvi et al., 2021). Previous studies demonstrated that intranasal Y1R agonist [Leu^31^, Pro^34^]NPY at 132 μg was adequate to counteract the depressive-like indicators after exposure to single prolonged stress (Serova et al., 2017). However, the equivalent dose was ineffective when performed on anxiety behaviour (Nwokafor et al., 2020). The requirement for higher amounts of intranasal Y1R agonist may be in part due to the degradation of the peptide by the dipeptidyl peptidase IV (DPP4), as occurs with NPY (Nahvi et al., 2021).

Verification of GALR2 contribution was also established while the GALR2 antagonist M871 blocked the boosted response observed, as previously described (Narvaez et al., 2015; Narvaez et al., 2016; Narvaez et al., 2018; Borroto-Escuela et al., 2021). Furthermore, neither M1145 and Y1R agonist nor their coadministration has shown locomotor alterations; thus, behavioural effects detected were independent of the motor actions, as described (Narvaez et al., 2015; Narvaez et al., 2016; Narvaez et al., 2018).

Interestingly, enhanced spatial learning in ageing rodents was recently demonstrated by genetically boosting neurogenesis in the dorsal hippocampal DG (Berdugo-Vega et al., 2020). With this approach, we observed the ability of the co-agonist treatment to enhance the cell proliferation in the DG of the dorsal hippocampus throughout 5-Bromo-2′-deoxyuridine (BrdU) expression analysis at 24 h. We have recently detected a rise of cell proliferation in the ventral hippocampus observed through BrdU analysis linked to antidepressant-like events (Borroto-Escuela et al., 2021). In agreement, the molecule P7C3, known to have neuroprotective actions, increased the neuronal cell proliferation associated with improved spatial learning and memory effects in neurodegenerative rodent models (Jiang et al., 2016; Loris et al., 2017).

The support of the cellular mechanisms was completed by considering the GALR2/Y1R heteroreceptor complexes upon agonist coactivation of both receptor protomers in Doublecortin-expressing neuroblasts. Thus, it is suggested that the mechanism of how the GALR2 and Y1R agonist coinjection triggers the neuroprotective actions is facilitated by the increase of GALR2/Y1R heteroreceptor complexes, leading to an enhancement of the neuronal proliferation in the dorsal DG upon their receptor protomer coactivation. GALR2/Y1R heteroreceptor complexes significantly increase their density, as established with PLA upon collective GALR2-Y1R agonists action in the sub-granular zone of the dentate gyrus in the dorsal hippocampus. The presence of GALR2/Y1R heteroreceptor complexes was validated in several regions, including the amygdala and the ventral hippocampus (Narvaez et al., 2015; Narvaez et al., 2016; Narvaez et al., 2018; Borroto-Escuela et al., 2021). Additionally, we confirmed how the 5HT1A-FGFR1 heterocomplex significantly stimulated hippocampal plasticity effects associated with hippocampal functional modifications (Borroto-Escuela et al., 2012; Narvaez et al., 2020).

The current data also support the hypothesis that the GALR2 and the Y1R agonist acting on the doublecortin (DCX)-cells expressing GALR2 and Y1R in the dorsal hippocampus established GALR2-Y1R heterocomplexes. Thus, agonists provoked allosteric GALR2-Y1R interaction could rise integration in the intracellular signalling, as we detected in the extracellular signal-regulated kinases (ERK) pathway through the SRE reporter assay (Borroto-Escuela et al., 2021).

Taken together, upon cotreatment with GALR2 and the Y1R agonist may promote the GALR2/Y1R heteroreceptor complexes configuration in the dorsal hippocampus linked to the improved spatial-memory effects observed. At the cellular level, these GALR2-Y1R heterocomplexes might induce the conversion of cell proliferation towards a neuronal lineage. Our results may postulate the beginning for developing heterobivalent agonist pharmacophores, targeting GALR2-Y1R heterocomplexes. It implicates especially the neuronal precursor cells of the dentate gyrus in the dorsal hippocampus for the novel management of neurodegenerative pathologies, as in Alzheimer’s disease.

## Data Availability

The original contributions presented in the study are included in the article/Supplementary Material, further inquiries can be directed to the corresponding author.
