# Supplementary material for: Intranasal Delivery of Galanin 2 and Neuropeptide Y1 Agonists Enhanced Spatial Memory Performance and Neuronal Precursor Cells Proliferation in the Dorsal Hippocampus in Rats
Source: Front Pharmacol. 2022 Feb 14;13:820210. doi: 10.3389/fphar.2022.820210 (PMC8893223; doi:10.3389/fphar.2022.820210)
Supplement: Supplementary file 1 [file DataSheet1.pdf]

Supplementary Material

Intranasal delivery of Galanin 2 and Neuropeptide Y Y1 agonists enhanced spatial memory performance and neuronal precursor cells proliferation in the dorsal hippocampus in rats

Dasiel O. Borroto-Escuela 1,2,3; Ramón Fores1,4; Mariana Pita 1,4,5; Miguel A. Barbancho 4; Pablo García-Zamorano 4; Natalia García Casares 4; Kjell Fuxe 1 & Manuel Narváez*1,4.

1 Department of Neuroscience, Karolinska Institute, Stockholm, Sweden,

2 Department of Biomolecular Science, Section of Physiology, University of Urbino, Urbino, Italy,

3 Grupo Bohío-Estudio, Observatorio Cubano de Neurociencias, Yaguajay, Cuba,

4 Instituto de Investigación Biomédica de Málaga, Facultad de Medicina, Universidad de Málaga, Málaga, Spain,

5 Departamento de Neurogenética. Instituto de Neurología y Neurocirugía. La Habana. Cuba.

*Correspondence:

Manuel Narváez, mnarvaez@uma.es

Intranasal administration of peptides

Galanin receptor 2 agonist (M1145), Y1R receptor agonist [Leu^{31}, Pro^{34}]NPY, GALR2 Antagonist M871 (Tocris Bioscience, Bristol, UK) were freshly dissolved in 20 µl distilled water. Each rat received 10 µl of them into each nostril with pipetteman and disposable plastic tip (1 mm in diameter) inserted no deeper than 1–1.5 mm into the nostril under light isoflurane anesthesia. Following the infusion, the head of the animal was held in a tilted back position for approximately 15 s to

prevent loss of solution from the nares. Y1 receptor agonist dose (132 µg) was chosen based on previous published dose-response curves. An equimolar dose for Galanin receptor 2 agonist was used (Serova et al., 2017; Serova et al., 2020).

Counting Procedure

BrdU-labeled cells number was counted with an Olympus BX51 microscope, Olympus, Denmark interfaced with a computer and a colour JVC digital video camera. For stereological analysis, sampling of BrdU positive cells was performed throughout the dentate gyrus of the dorsal hippocampus in the rostrocaudal dimension using the optical fractionator. This method combines the optical dissector with a fractionator sampling scheme to exclude volume divergences (Gundersen et al., 1988). Counterstaining with phase contrast allowed delineation of different areas in each section (Paxinos and Watson, 1986). Numbers of BrdU positive cells were quantified in at least eight representative 150 µm, evenly spaced sections per animal (4 rats per group). A random set of sampling frames with a known area (α frame) was generated for each section using the C.A.S.T. Grid (Olympus; Albertslund, Denmark). After the objects were counted (ΣQ-) the total number of positive cells were estimated as: N = ΣQ- x fs x fa x fh (Gundersen et al., 1988), where fs is the numerical fraction of the section used, fa is the areal fraction and fh is the linear fraction of section thickness. The quantification was limited to the granular cell layer and subgranular zone. Subgranular region was outlined as a band-limited by three nuclei down from the edge between the granular cell layer and the hilar region, and cells located more than two cells away from the subgranular zone were excluded. The coefficient of error (CE) for each estimation and animal ranged from 0.05 to 0.1. The total CE of each group ranged from 0.07 to 0.08. Counting of labelled cells was set starting at 5 µm below the surface and focusing through the 20 µm section optical plane, and the number of counting frames used was 90-110 per animal. We have used this stereological procedure is previous studies (Narvaez et al., 2016;Narvaez et al., 2018).

Supplementary Figure 1

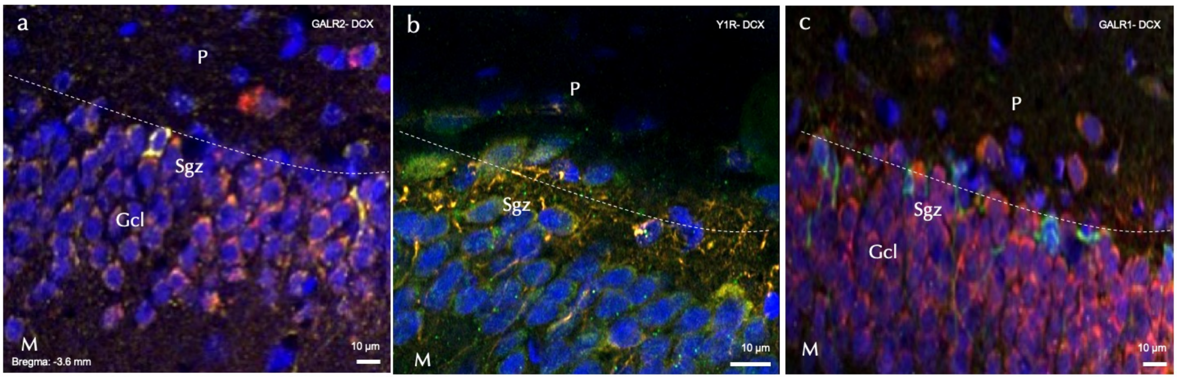

Supplementary figure 1. Representative laser-scanned confocal micrographs illustrate the polymorphic (P), granular (Gcl), and molecular (M) layers of the dorsal dentate gyrus (Bregma: –3.6 mm). Nuclei are shown in blue (DAPI). **(a)** Laser scanned confocal micrographs demonstrating the neuronal colocalization (yellow) of endogenous Galanin receptor subtype 2 (GALR2) (red)(Alomone, Rabbit, 1:100) and Doublecortin (green, microtubule-associated protein(Gleeson JG, 1999))(C-18, Santa Cruz, 1:500) in the subgranular zone (Sgz) of the dentate gyrus (defined as a band-limited by three nuclei down from the apparent border between the Gcl and the Pol region). **(b)** Laser scanned confocal micrographs demonstrating the colocalization (yellow) of endogenous Neuropeptide Y1R (green)(Santa Cruz, Goat, 1:200) and Doublecortin (red)(Abcam, Rabbit, 1:500) in the Sgz of the dentate gyrus. **(c)** Laser scanning confocal micrographs are demonstrating that endogenous Galanin receptor subtype 1 (GALR1)(red)(Alomone, Rabbit, 1:100) and Doublecortin (green)(C-18, Santa Cruz, 1:500) do not colocalize in the Sgz of the dentate gyrus.

References

Gundersen, H.J., Bagger, P., Bendtsen, T.F., Evans, S.M., Korbo, L., Marcussen, N., Moller, A., Nielsen, K., Nyengaard, J.R., Pakkenberg, B., and Et Al. (1988). The new stereological tools: disector, fractionator, nucleator and point sampled intercepts and their use in pathological research and diagnosis. APMIS 96, 857-881.

Narvaez, M., Borroto-Escuela, D.O., Millon, C., Gago, B., Flores-Burgess, A., Santin, L., Fuxe, K., Narvaez, J.A., and Diaz-Cabiale, Z. (2016). Galanin receptor 2- neuropeptide Y Y1 receptor interactions in the dentate gyrus are related with antidepressant-like effects. Brain Struct Funct 221, 4129-4139.

Narvaez, M.; Borroto-Escuela, D.O.; Santin, L.; Millon, C.; Gago, B.; Flores-Burgess, A.; Barbancho, M.A.; Perez de la Mora, M.; Narvaez, J.; Diaz-Cabiale, Z.; et al. A Novel Integrative Mechanism in Anxiolytic Behavior Induced by Galanin 2/Neuropeptide Y Y1 Receptor Interactions on Medial Paracapsular Intercalated Amygdala in Rats. *Front Cell Neurosci* **2018**, *12*, 119, doi:10.3389/fncel.2018.00119.

Paxinos, G. and Watson, C. (1986) The Rat Brain in Stereotaxical Coordinates. Second edition. Academic Press, San Diego.

Serova, L.; Mulhall, H.; Sabban, E. NPY1 Receptor Agonist Modulates Development of Depressive-Like Behavior and Gene Expression in Hypothalamus in SPS Rodent PTSD Model. *Front Neurosci* **2017**, *11*, 203, doi:10.3389/fnins.2017.00203.

Serova, L.I.; Hansson, E.; Sabban, E.L. Effect of intranasal administration of neuropeptide Y and single prolonged stress on food consumption and body weight in male rats. *Neuropeptides* **2020**, *82*, 102060, doi:10.1016/j.npep.2020.102060.
